# Self-Healing Bilayer Hydrogel Solid-State Electrochemical Platform: Time-Resolved In Situ Dynamic Monitoring of *Escherichia coli* Activity

**DOI:** 10.3390/gels12060538

**Published:** 2026-06-15

**Authors:** Ye Li, Chaofan Zhang, Miao Zhang, Shi Zhou, Yanping Yu, Xiaoyan Yu, Ximing Cui, Xiangge Qin

**Affiliations:** 1 College of Materials Science and Engineering Technology, Jiamusi University, Jiamusi 154007, China; liye881006@163.com (Y.L.); yuxiaoyan@jmsu.edu.cn (X.Y.); 2 College of Pharmacy, Jiamusi University, Jiamusi 154007, China; zhous146@nenu.edu.cn; 3 Jiamusi Center for Disease Control and Prevention, Jiamusi 154007, China; 15046463695@163.com

**Keywords:** self-healing hydrogel, bilayer electrochemical platform, in situ metabolite monitoring, microbial activity detection, antibiotic susceptibility evaluation

## Abstract

Achieving in situ and time-resolved monitoring of microbial metabolites without disrupting the microbial growth environment remains a key challenge in electrochemical biosensing. Herein, we propose a self-healing bilayer hydrogel-based solid-state electrochemical sensing platform for the in situ, time-resolved analysis of purine metabolites produced by *Escherichia coli* (*E. coli*). This platform integrates an upper Agar culture module and a lower borax-crosslinked poly(vinyl alcohol) (PVA) detection module, forming a contiguous structure that allows metabolites (e.g., guanine, xanthine, hypoxanthine) to migrate across the solid–solid interface for sensitive electrochemical detection. The detection layer exhibits excellent ionic conductivity; when coupled with its robust structural self-healing capacity, the platform achieved a detection limit of 0.05 µM for guanine. For *E. coli* detection, a linear response range of 1.1 × 10^6^ to 9.5 × 10^6^ CFU·mL^−1^ (R^2^ = 0.9974) was obtained, and relative standard deviations (RSDs) of less than 2.34% even after two weeks of storage. Leveraging this integrated design, the platform enables continuous, label-free tracking of bacterial metabolic dynamics throughout all growth phases. Notably, it detects metabolic transition points earlier than traditional plate counting methods and accurately evaluates antibiotic inhibition trends, with results consistent with colony-forming unit (CFU) analysis. This integrated culture–detection architecture thus provides a versatile strategy for functional microbial analysis and rapid antimicrobial susceptibility testing.

## 1. Introduction

Accurate characterization of microbial metabolic activity is paramount for environmental remediation, clinical diagnostics [1,2], food safety, and antibiotic efficacy evaluation [3,4,5,6]. Conventional microbial assessment techniques, such as microscopic counting, plate counting [7,8], and turbidimetric measurements, primarily rely on cell density or colony formation as proliferation-based indicators [9,10,11]. However, these methods provide only an indirect reflection of microbial growth and fail to reveal the dynamic evolution of microbial viability in response to environmental stimuli or pharmacological interventions [12]. In fact, microbial activity is reflected not only by the growth rate but also profoundly by the metabolic intensity [13,14,15]. Therefore, there is an urgent need for technologies capable of directly interpreting metabolic processes to achieve a comprehensive activity assessment [16,17]. Electrochemical analysis has attracted increasing attention for microbial monitoring due to its high sensitivity, operational simplicity [18,19], rapid response [20,21], and compatibility with miniaturized integration. Among various electrochemical strategies [22,23,24], directly targeting microbial metabolites (e.g., purine compounds) offers a more straightforward readout of the metabolic state [25,26,27,28,29]. Recently, several electrochemical platforms have achieved highly sensitive detection of purine metabolites [30]. For instance, membrane-modified glassy carbon electrodes enable the simultaneous detection of uric acid and xanthine [31], while reduced graphene oxide–poly(L-glutathione) composite electrodes allow for the independent detection of multiple purine derivatives with excellent stability [32,33,34]. However, most of these methods require extraction, dissolution [35], or sample transfer into a homogeneous liquid phase [36,37,38,39]. Such requirements preclude the time-resolved monitoring of microbial growth in solid media and expose labile metabolites to atmospheric oxygen, potentially leading to signal attenuation [40,41].

To address these challenges, various in situ electrochemical strategies have been developed. For example, microelectrode arrays have been employed to probe local metabolic signals, but prolonged measurements often disturb the biological growth process. In contrast, platforms such as light-addressable potentiometric sensors (LAPS), field-effect transistors (FETs) [42,43], and microfluidic systems can monitor metabolic signals in real time [42]. Nevertheless, a fundamental bottleneck remains: the temporal and operational decoupling of cultivation and detection. Achieving true in situ detection at the native growth site without disturbing the biological rhythm remains a significant challenge [44,45]. Currently, methods capable of achieving the seamless simultaneity of bacterial growth and electrochemical monitoring within a single, undisturbed system remain limited [46,47,48].

**Scheme 1:**
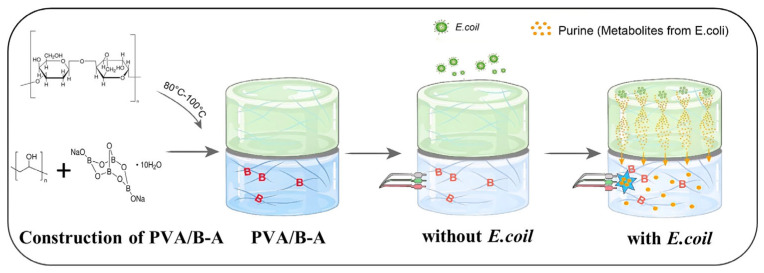
Schematic diagram of the construction of the PVA/B-A bilayer hydrogel platform for time-resolved in situ dynamic monitoring of *E. coli* activity.

In this work, we propose a self-healing PVA/B–A bilayer hydrogel platform for the synchronized integration of microbial proliferation and electrochemical metabolic interrogation under solid-state cultivation conditions. The hallmark of this design lies in its support for a “growth-while-detecting” function through a functionally compartmentalized yet physically continuous bilayer architecture. Specifically, the upper Agar-based module serves as a native solid-state cultivation zone for *E. coli* proliferation, while the lower borax-crosslinked poly(vinyl alcohol) hydrogel acts as an ionically conductive and self-healing electrochemical sensing zone. Purine-type metabolites secreted by bacteria can spontaneously diffuse across the continuous solid–solid hydrogel interface from the cultivation layer to the detection layer, allowing time-resolved electrochemical readout of bacterial metabolic activity without liquid-phase extraction, sample transfer, external labeling, or disruption of the native growth microenvironment. Compared with previously reported hydrogel-based or solid-state electrochemical microbial sensing systems, the present platform provides several distinctive features: spatial separation but functional coupling of cultivation and sensing, non-invasive metabolite communication across a solid–solid hydrogel interface, and self-healing recovery of the sensing matrix after repeated electrode insertion. These features distinguish the PVA/B–A platform from conventional hydrogel sensors, microelectrode arrays, LAPS/FET-based devices, and microfluidic systems, many of which rely on liquid-phase sampling, external perfusion, direct electrode contact with the culture region, or partial temporal/operational decoupling between cultivation and detection. By enabling continuous and label-free monitoring of bacterial metabolic dynamics at the native growth site, this bilayer hydrogel architecture establishes a solid-state “growth-while-detecting” strategy for functional microbial analysis and rapid antimicrobial susceptibility evaluation (Scheme 1).

## 2. Results and Discussion

### 2.1. Characterization of the Self-Healing PVA/B Hydrogel Detection

**Figure 1:**
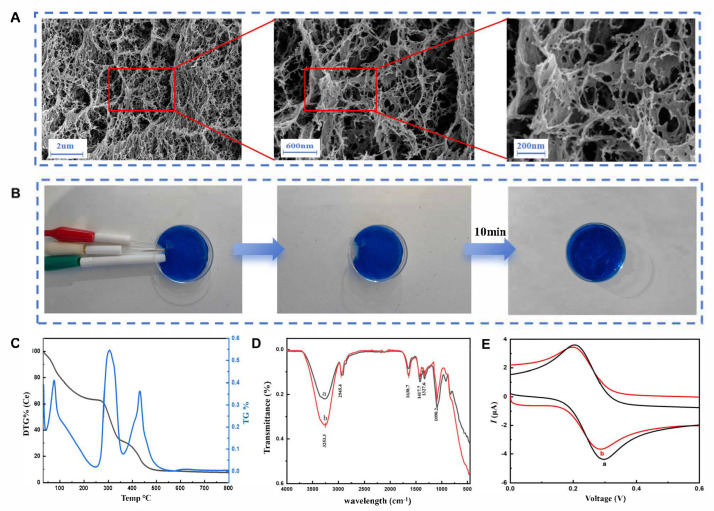
Characterization of the self-healing PVA/B hydrogel. (**A**) SEM images of PVA/B. (**B**) Self-healing behavior of the PVA/B hydrogel. (**C**) TGA profile of PVA/B. (**D**) FTIR spectra of (a) PVA and (b) borax-crosslinked PVA (PVA/B). (**E**) Cyclic voltammograms (CVs) of K_3_[Fe(CN)_6_]/K_4_[Fe(CN)_6_] recorded in (a) aqueous solution and (b) within the PVA/B hydrogel. Concentration of K_3_[Fe(CN)_6_]/K_4_[Fe(CN)_6_] mixture: 1 mM; concentration of KCl: 0.1 M; scan rate: 90 mV·s^−1^; accumulation time: 270 s; accumulation potential: 0 V; working electrode: glassy carbon electrode (GCE).

As the solid-state electrochemical detection layer, the borax-crosslinked PVA hydrogel (PVA/B) was systematically characterized in terms of its structural and functional properties. SEM images (Figure 1A) reveal a continuously interconnected porous architecture with pore sizes of approximately 300–500 nm. This nanoscale porous network helps maintain the mechanical stability of the hydrogel while facilitating ion and metabolite diffusion within the solid matrix, which is essential for rapid mass transport and stable electrochemical responses under solid-state conditions [49,50,51]. On this basis, the self-healing ability of the material under practical solid-state measurement conditions was further examined. As shown in Figure 1B, the PVA/B hydrogel was dyed blue for visual clarity. After electrode insertion and removal, the hydrogel restores its overall structure within 10 min without external stimulation. This self-healing behavior originates from the reversible borate-ester crosslinked network [52,53], enabling the sensing interface to maintain continuity and stability during repeated in situ measurements. 

Thermogravimetric analysis (Figure 1C) indicates that the crosslinked hydrogel exhibits no obvious weight loss below 150 °C, demonstrating sufficient thermal and structural stability under electrochemical testing conditions. The PVA sample (a in Figure 1D) exhibits characteristic peaks at 3253 cm^−1^ (O-H stretching), 2938 cm^−1^ (C-H stretching), 1648 cm^−1^ (O-H bending), and 1089 cm^−1^ (C-O stretching). In comparison, the PVA/B sample (b in Figure 1D) shows a distinct change in the C–O stretching vibration peak near 1090 cm^−1^, confirming the formation of a dynamic borate ester crosslinking network.

Using the classical redox probe K_3_[Fe(CN)_6_]/K_4_[Fe(CN)_6_], the PVA/B hydrogel (b in Figure 1E) maintained well-defined redox peaks, with electrochemical responses comparable to those observed in aqueous solution (a in Figure 1E) [52,53,54,55]. The ion-transport and interfacial charge-transfer properties were further evaluated by EIS (Figure S6). These results indicate that the hydrogel network introduces additional ionic, charge-transfer, and diffusion resistance compared with the aqueous system. Nevertheless, the stable CV and EIS responses confirm that the PVA/B hydrogel maintains continuous hydrated ion-transport pathways and supports effective interfacial redox reactions under solid-state conditions.

In summary, the PVA/B hydrogel integrates a robust porous architecture and rapid self-healing ability with excellent ionic conductivity. These structural features not only ensure efficient mass transport and electron transfer, but also guarantee the structural integrity and stability of the interface. Consequently, the PVA/B hydrogel serves as an ideal solid-state electrochemical sensing platform that is well-suited for reliable and repeated measurements.

### 2.2. Construction and Electrochemical Optimization of the PVA/B-A Bilayer Platform

**Figure 2:**
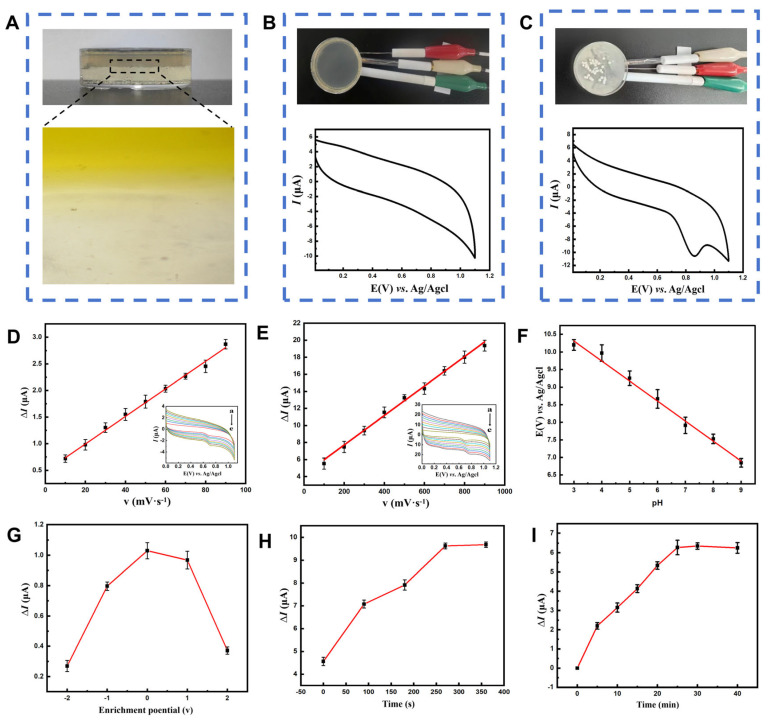
Electrochemical response and condition optimization of the PVA/B-A bilayer platform. (**A**) Construction of PVA/B-A. (**B**) CV behavior of PVA/B-A without *E. coli* inoculation. (**C**) CV behavior of PVA/B-A after *E. coli* inoculation. (**D**) Effect of slow scan rates on guanine CV in PVA/B-A; inset: CVs at different scan rates (a→e): 10, 20, 30, 40, 50, 60, 70, 80, 90, 100 mV·s^−1^; (**E**) Effect of fast scan rates on guanine CV in PVA/B-A; inset: CVs at different scan rates (a→e): 100, 200, 300, 400, 500, 600, 700, 800, 900, 1000 mV·s^−1^. Guanine concentration in all cases: 2 × 10^−5^ mol·L^−1^. (**F**) Effect of pH on guanine CV peak current in PVA/B-A; scan rate: 90 mV·s^−1^; accumulation time: 270 s; accumulation potential: 0 V; working electrode: GCE; guanine concentration: 1 × 10^−5^ mol·L^−1^. (**G**) Effect of accumulation potential on guanine CV peak current in PVA/B-A; scan rate: 90 mV·s^−1^; accumulation time: 0 s. (**H**) Effect of accumulation time on guanine CV peak current in PVA/B-A; scan rate: 90 mV·s^−1^; accumulation potential: 0 V. (**I**) Effect of diffusion time on guanine CV peak current in PVA/B-A; scan rate: 90 mV·s^−1^; accumulation time: 270 s; accumulation potential: 0 V; working electrode: GCE; guanine concentration: 2.5 × 10^−5^ mol·L^−1^.

To evaluate the feasibility of the PVA/B-A bilayer detection system for in situ monitoring, its structural configuration and interfacial microfeatures were first examined (Figure 2A). Microscopic imaging reveals a well-defined, continuous, and stable physical interface between the upper Agar cultivation module and the lower PVA/B detection module. Owing to the abundance of hydroxyl (–OH) groups in both PVA and Agar molecular chains, the two hydrogel layers are tightly integrated at the interface primarily through strong hydrogen-bonding interactions and physical chain entanglement. In addition, the PVA/B hydrogel, constructed from a dynamic borate ester-crosslinked three-dimensional network, provides structural support for robust interfacial adhesion across the solid–solid boundary [56,57,58].

The background electrochemical behavior of the blank system was evaluated by cyclic voltammetry (CV) (Figure 2B). In the absence of bacterial inoculation, direct insertion of the three-electrode system into the detection module resulted in only negligible background currents, indicating excellent electrochemical inertness of the PVA/B hydrogel matrix. In contrast, after *E. coli* inoculation and cultivation (Figure 2C), a pronounced oxidation peak emerged in the potential range of +0.80 to +0.90 V, demonstrating that bacterial metabolites can efficiently migrate across the solid–solid interface and be electrochemically detected [59,60,61]. In addition, a purine-related electrochemical signal was also detected from Staphylococcus aureus, a representative Gram-positive bacterium (Figure S4), indicating the potential applicability of the bilayer hydrogel platform to different bacterial species.

To elucidate the controlling mechanism of purine electrochemical oxidation in the PVA/B-A detection system, the dependence of the cyclic voltammetric peak current (Iₚ) on scan rate was systematically investigated. In the low scan-rate regime, as shown in Figure 2D, the peak current exhibited a linear relationship with the square root of the scan rate (Iₚ = 0.276 ν1/2 − 0.153, R^2^ = 0.9912), indicating a diffusion-controlled process. The calculated diffusion coefficient (D ≈ 1.3 × 10^−6^ cm^2^·s^−1^) is comparable to that in aqueous systems (5.0–8.0 × 10^−6^cm^2^s^−1^), demonstrating that the PVA/B network provides efficient mass transport pathways even under solid-state conditions—an essential prerequisite for time-resolved signal response.

At higher scan rates (Figure 2E), the peak current scaled linearly with the scan rate (I_p_ = 0.0172 ν + 3.804, R^2^ = 0.9932), suggesting a transition to an adsorption-controlled regime. Based on the adsorption-controlled model, the surface coverage of purine on the electrode (Γ* ≈ 4.8 × 10^−10^ mol·cm^−2^) was estimated, which is comparable to monolayer adsorption capacity. Further analysis using the Laviron equation to fit the E_p_–ln ν relationship yielded a charge-transfer coefficient α ≈ 0.52 and a heterogeneous electron-transfer rate constant k_s_ ≈ 1.1 × 10^−2^ s^−1^, indicating that interfacial electron-exchange kinetics remain highly efficient within the PVA/B hydrogel environment.

The dependence of the guanine oxidation peak potential on pH was further examined (Figure 2F), yielding the linear relationship E_p_ (V) = 1.218 − 0.0560 pH (R^2^ = 0.9916). The slope of −56.5 mV·pH^−1^ is close to the theoretical value of −59 mV·pH^−1^, confirming that the purine oxidation process follows a two-proton/two-electron proton-coupled electron-transfer (PCET) mechanism. This feature enables the electrochemical signal to sensitively respond to microenvironmental acidification induced by bacterial metabolism.

Further optimization experiments were conducted to determine the optimal accumulation potential and accumulation time. As shown in Figure 2G, the purine oxidation peak current reached its maximum at an accumulation potential of 0 V. Further optimization at 0 V showed that the peak current increased with accumulation time and plateaued after approximately 270 s (Figure 2H). Therefore, 0 V and 270 s were selected as the optimal accumulation potential and time, respectively. In addition, diffusion time analysis (Figure 2I) revealed a rapid increase in signal intensity during the initial stage, followed by attainment of a dynamic diffusion equilibrium after approximately 30 min, at which point the system entered a steady-state detection regime. These results indicate that the platform is well suited not only for rapid detection but also for stable and continuous monitoring.

### 2.3. Electrochemical Signal Assignment of Bacterial Metabolites in the PVA/B-A Platform

**Figure 3:**
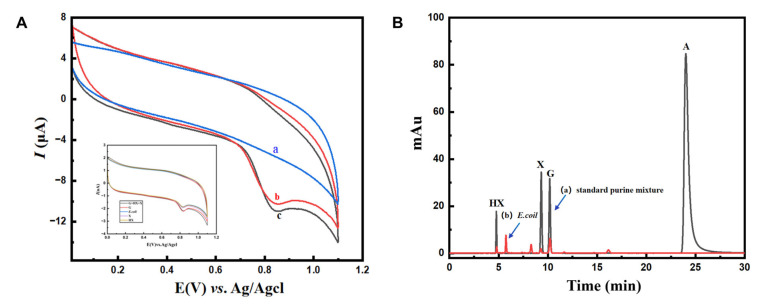
Electrochemical signal assignment of *E. coli* in the PVA/B-A system. (**A**) Comparison of CV responses between purine standards: (a) blank PVA/B-A system without inoculation; (b) metabolic products of *E. coli* in PVA/B-A; (c) purine standard mixture in PVA/B-A. Insets: standard-addition CV responses obtained by spiking HX, X, G, and their mixture into the actual *E. coli* metabolic matrix. Concentrations of individual purine standards and the mixture: 2 × 10^−5^ mol·L^−1^. (**B**) Chromatograms of *E. coli* metabolic products in PVA/B-A and purine standard mixtures (xanthine, guanine, adenine/hypoxanthine). *E. coli* concentration: 1.1 × 10^9^ CFU·mL^−1^; purine standard mixture concentration: 1.6 × 10^−5^ mol·L^−1^; pH ≈ 6. Scan rate: 90 mV·s^−1^; accumulation time: 270 s; accumulation potential: 0 V; working electrode: GCE.

To elucidate the origin of the electrochemical signals observed in the PVA/B-A system, the electrochemical behaviors of *E. coli* metabolites and purine standards were systematically compared. As shown in the main panel of Figure 3A, the blank PVA/B-A system without *E. coli* inoculation (a in Figure 3A) exhibited only background currents within the tested potential window. After *E. coli* inoculation and cultivation, when the terminal culture pH reached approximately 6, the metabolic products of *E. coli* (b in Figure 3A) generated a single, well-defined oxidation peak in the range of 0.80–0.90 V. This peak position closely matched that of the purine standard mixture (X + HX + G) recorded at pH ≈ 6 (c in Figure 3A). These results suggest that the electrochemical signal detected by the PVA/B–A platform is closely associated with purine-type metabolites secreted by *E. coli*. To further strengthen this assignment in the actual bacterial matrix, standard-addition experiments were subsequently performed.

To further validate the contribution of individual purine metabolites in the actual bacterial matrix, standard-addition experiments were performed by spiking guanine (G), xanthine (X), and hypoxanthine (HX) into the *E. coli*-derived metabolic matrix in the PVA/B–A platform. As shown in the inset of Figure 3A, the original *E. coli* metabolic matrix exhibited a purine-related oxidation peak current of 1.869 μA. After the addition of HX, X, and G, the peak currents increased to 1.879, 1.934, and 2.214 μA, respectively, corresponding to current increments of 0.010, 0.065, and 0.345 μA. These results indicate that G produced the most pronounced enhancement of the oxidation signal, while X and HX contributed much weaker responses under the same conditions. Notably, the mixed addition of G, X, and HX yielded a peak current of 2.218 μA, which was very close to that obtained after G addition alone. Therefore, the oxidation peak at +0.80–0.90 V can be assigned to the collective oxidation of purine-type metabolites in the bacterial matrix, with guanine serving as the dominant contributor under the present experimental conditions.

High-performance liquid chromatography (HPLC) analysis was further employed to validate the assignment of the electrochemical signals. As shown in Figure 3B, hypoxanthine, xanthine, and guanine were simultaneously identified in the metabolic products of *E. coli* cultivated in the PVA/B-A system. Their retention times (6.48 min, 9.29 min, and 10.83 min, respectively) closely matched those of the corresponding standards (6.52 min, 9.35 min, and 10.88 min) [62,63,64]. These results confirm that the metabolic profile of *E. coli* under solid-state cultivation conditions comprises multiple purine-type metabolites.

Although multiple purine metabolites contribute collectively to the overall electrochemical response, the standard-addition results indicate that guanine dominates the oxidation signal under the experimental conditions used in this study. Accordingly, the oxidation peak at +0.80–0.90 V is more appropriately regarded as a guanine-dominated purine-related signal rather than a response from a single metabolite. This signal can therefore serve as a functional indicator of the overall purine metabolic level and reflect the metabolic activity of *E. coli* during solid-state cultivation. In addition, the mildly acidic microenvironment induced by bacterial metabolism may cause a slight shift in the oxidation potential, which is consistent with the proton-coupled electron-transfer behavior of purine oxidation [65,66,67].

### 2.4. Quantitative Performance of the PVA/B-A Platform for Bacterial Detection

**Figure 4:**
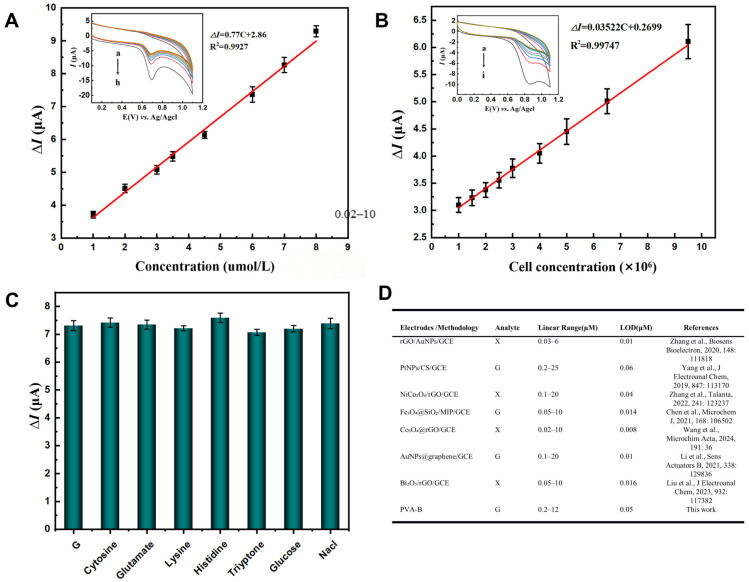
Quantitative relationship between *E. coli* cell concentration and electrochemical signal intensity in the PVA/B-A system. (**A**) Linear correlation between current response and guanine (G) concentration in PVA/B-A; inset: CVs recorded at different G concentrations. G concentrations (a→i): 1.0, 2.0, 3.0, 3.5, 4.5, 6.0, 7.0, and 8.0 μM. (**B**) Linear correlation between *E. coli* cell concentration and electrochemical signal intensity; inset: CVs obtained at different *E. coli* concentrations. *E. coli* cell numbers (a→j): 1.1 × 10^6^, 1.3 × 10^6^, 1.9 × 10^6^, 2.5 × 10^6^, 2.9 × 10^6^, 3.9 × 10^6^, 5.1 × 10^6^, 6.4 × 10^6^, and 9.5 × 10^6^ CFU·mL^−1^. (**C**) Anti-interference performance of the PVA/B-A system. (**D**) Comparison of different electrochemical methods for the detection of G and X. Scan rate: 90 mV·s^−1^; accumulation time: 270 s; accumulation potential: 0 V [68,69,70,71,72].

Without the need for sample extraction or liquid-phase conversion, the analytical performance of the PVA/B-A bilayer platform under solid-state cultivation conditions was systematically evaluated. Using guanine as a representative purine metabolite (Figure 4A), a stable oxidation response was observed in the potential range of +0.80–0.90 V. The change in peak current (ΔI) exhibited a good linear correlation with guanine concentration over the range of 1.0–8.0 μM, yielding a linear regression equation of ΔI (μA) = 0.77 C + 2.86 with a correlation coefficient of R^2^ = 0.9927. The limit of detection (S/N = 3) was calculated to be 0.05 μM, which is comparable to those reported for related sensors in the literature (Figure 4D). Furthermore, the system demonstrated excellent reproducibility and repeatability, with relative standard deviations (RSDs) of 2.11% and 1.77%, respectively (Figure S1A,B). After storage at 4 °C for two weeks, the purine signal in the hydrogel detection layer remained stable (RSD = 2.34%, Figure S2), indicating that the three-dimensional hydrogel network effectively immobilizes and protects purine metabolites. Moreover, the hydrogel limits the purine exposure to atmospheric oxygen during in situ detection, and thereby retards air-induced, non-electrochemical oxidation prior to electrochemical interrogation, resulting in improved signal stability.

More importantly, the platform enabled a direct correlation between electrochemical signal intensity and *E. coli* cell concentration under solid-state cultivation conditions. As shown in Figure 4B, a pronounced linear relationship was observed between the metabolite-derived electrochemical signal and bacterial concentration over the range of 1.1–9.5 × 10^6^ CFU·mL^−1^, yielding a linear regression equation of ΔI = 0.03 C + 0.26 with a correlation coefficient of R^2^ = 0.9974. The corresponding limit of detection was determined to be 0.3 × 10^6^ CFU·mL^−1^, indicating that the platform enables quantitative evaluation of bacterial metabolic activity during solid-state cultivation, Within the detection range, no obvious saturation phenomenon was observed, and a linear relationship was maintained throughout. To further clarify the analytical characteristics of the PVA/B–A platform, a comparison with recent electrochemical bacterial sensors is provided in Table S2. Many reported methods have achieved rapid and sensitive bacterial detection in liquid-phase samples after appropriate sample processing or transfer. In contrast, the present platform is designed for in situ monitoring under solid-state cultivation conditions, allowing bacterial metabolites to be detected without liquid extraction or disruption of the growth microenvironment. This feature suggests that the platform is suitable for time-resolved metabolic monitoring in solid-state bacterial culture systems.

Anti-interference experiments further demonstrated the selectivity of the PVA/B-A system (Figure 4C). The responses to common amino acids (such as glutamic acid, lysine, and histidine), other nucleobases, and culture-medium components all resulted in signal variations below 4%, indicating that the electrochemical response predominantly originates from purine-type metabolites. Moreover, the recovery rates ranging from 96.0% to 101.5% for guanine in *E. coli* samples further confirmed the analytical reliability and accuracy of the PVA/B-A platform for in situ quantification of guanine under solid-state cultivation conditions (Table S1). Notably, all of the above analytical performances were achieved directly under solid-state cultivation conditions, without relying on liquid-phase sample transfer or pretreatment procedures. This distinctive feature enables the PVA/B-A platform to reflect changes in bacterial activity at an earlier stage and in a more functionally relevant manner while preserving the native growth state of the bacteria, thereby fundamentally distinguishing it from conventional liquid-phase electrochemical sensing strategies.

### 2.5. Time-Resolved Monitoring of Bacterial Growth Using the PVA/B-A Platform

**Figure 5:**
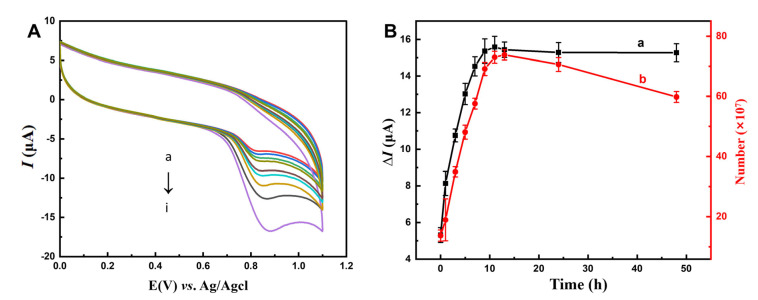
Time-resolved monitoring of *E. coli* growth using the PVA/B-A platform. (**A**) Current responses of PVA/B-A recorded at different incubation times. Incubation times (a→i): 1, 3, 5, 7, 9, 11, 13, 24, and 48 h; initial inoculation concentration: 75 × 10^6^ CFU·mL^−1^. (**B**) Growth curves of *E. coli*. (a) Electrochemical detection using the PVA/B-A platform. (b) Plate counting method; initial inoculation concentration: 75 × 10^6^ CFU·mL^−1^. Scan rate: 90 mV·s^−1^; accumulation time: 270 s; accumulation potential: 0 V.

To validate the capability of the PVA/B-A bilayer sensing platform for dynamic metabolic monitoring, continuous in situ electrochemical measurements of *E. coli* growth were carried out over a 50 h period using the PVA/B-A system (Figure 5A). The results show that the oxidation peak current associated with purine metabolism exhibited distinct, stage-dependent variations over time, effectively reflecting the dynamic evolution of bacterial metabolic activity during different growth phases. During the initial incubation stage (1–9 h), the peak current increased rapidly, corresponding to the transition of bacteria from the lag phase to the exponential growth phase, during which metabolic activity often intensifies prior to a pronounced increase in cell number. Between 9 and 13 h, the growth of the electrochemical signal gradually slowed, indicating a transition toward the stationary phase. After 13 h, the peak current continuously declined, consistent with metabolic attenuation and cell death induced by nutrient depletion and accumulation of metabolic waste products.

Compared with the growth curve obtained by the plate counting method (Figure 5B), both approaches exhibited consistent overall trends; however, the PVA/B-A platform (a in Figure 5B) reflected bacterial metabolic transitions at earlier time points. In the plate counting curve (b in Figure 5B), the corresponding transition toward the stationary phase was mainly observed at approximately 13 h, while the decline tendency became evident at approximately 24 h. Thus, compared with the plate counting method, the electrochemical platform reflected these two transitions approximately 4 h and 11 h earlier, respectively. This demonstrates that electrochemical signals based on purine metabolism can reflect changes in the functional state of bacteria earlier than conventional colony-formation-based counting methods. By enabling continuous, label-free monitoring directly under solid-state cultivation conditions, the PVA/B-A platform offers a distinct advantage for time-resolved evaluation of bacterial activity and growth kinetics.

### 2.6. Evaluation of Antibiotic Inhibition Using the PVA/B-A Platform

**Figure 6:**
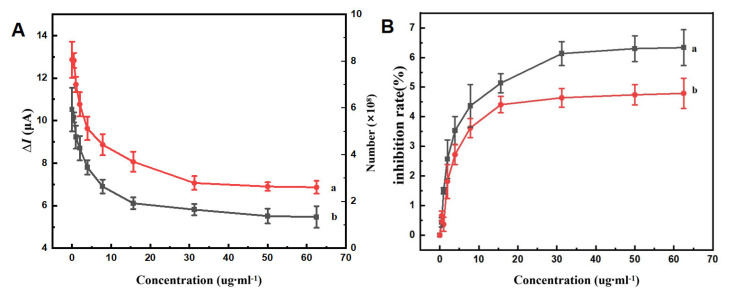
(**A**) Effect of different doses of levofloxacin on *E. coli* peak current intensity and bacterial counts (*n* = 3). (**B**) Effect of different doses of levofloxacin on the inhibition rate of (*n* = 3): a, plate counting method; b, purine CV peak intensity in the bilayer detection system. Levofloxacin concentration range: 0–64 μg·mL^−1^; *E. coli* inoculation concentration: 75 × 10^6^ CFU·mL^−1^. Working electrode: GCE; Scan rate: 90 mV·s^−1^; accumulation time: 270 s; accumulation potential: 0 V.

To validate the functional capability of the PVA/B-A platform for antibiotic susceptibility assessment, the effects of levofloxacin at different concentrations on the metabolic activity of *E. coli* were systematically investigated under solid-state cultivation conditions. As a typical concentration-dependent quinolone antibiotic [73,74,75], levofloxacin exerts its antibacterial activity by inhibiting bacterial DNA gyrase and topoisomerase IV, thereby blocking DNA replication and transcription processes and ultimately leading to a pronounced reduction in bacterial metabolic activity [76,77] (Figure 6).

The experimental results indicate that, as the concentration of levofloxacin increased from 0 to 32 μg·mL^−1^, the electrochemical oxidation peak current associated with purine metabolism decreased continuously, exhibiting a clear concentration-dependent inhibitory trend. When the drug concentration was further increased beyond 32 μg·mL^−1^, the electrochemical signal gradually approached a plateau, suggesting that bacterial metabolic activity had been suppressed to near-maximum levels. This trend was in good agreement with the colony-forming unit (CFU) results obtained by the plate counting method, with both approaches displaying pronounced inhibition enhancement and plateau behavior over the same concentration range. Inhibition rates calculated using the two methods showed that, at 32 μg·mL^−1^, the maximum inhibition rate determined by plate counting was approximately 69.5%, while that derived from purine-based electrochemical signals was about 58.0%. Despite the difference in absolute values, the two methods exhibited good consistency in terms of overall inhibition trends.

Compared with conventional methods that rely on prolonged cultivation and colony formation, the PVA/B-A platform enables time-resolved capture of antibiotic-induced metabolic inhibition directly under solid-state cultivation conditions, without the need for sample separation or additional pretreatment steps. By preserving the native growth state of bacteria, the platform substantially shortens the detection time while providing functionally relevant information on metabolic activity. These results not only validate the practical applicability of the PVA/B-A system for evaluating antibiotic efficacy and bacterial activity, but also demonstrate that its bilayer solid-state architecture holds strong potential for extension to other bacterial systems and metabolic pathways. As such, this approach offers a versatile biosensing strategy for drug susceptibility and pharmacodynamic assessment in solid-state microbial systems.

## 3. Conclusions

In this study, a self-healing PVA/B–A bilayer hydrogel solid-state electrochemical platform was developed for in situ and time-resolved monitoring of *E. coli* metabolic activity. The platform integrated an Agar cultivation layer with a borax-crosslinked PVA hydrogel detection layer, enabling purine-type metabolites secreted by *E. coli* to diffuse across the solid–solid interface for electrochemical detection. The oxidation signal was assigned to guanine-dominated purine metabolites and was used to evaluate bacterial metabolic activity under solid-state cultivation conditions. Time-resolved measurements showed that the platform detected bacterial metabolic transition points earlier than the plate counting method. Levofloxacin inhibition experiments further confirmed that the electrochemical response reflected antibiotic-induced changes in *E. coli* metabolic activity, with trends consistent with CFU analysis. Overall, the proposed bilayer hydrogel system offers a scalable platform concept for time-resolved microbial metabolic analysis and rapid antimicrobial susceptibility assessment.

## 4. Materials and Methods

### 4.1. Materials

Guanine (G), xanthine (X), hypoxanthine (HX), adenine, cytosine, glutamic acid, lysine, and histidine were purchased from Sigma-Aldrich (St. Louis, MO, USA). Nutrient agar medium and tryptic soy broth were obtained from Beijing Sanyao Science & Technology Development Co., Ltd. (Beijing, China). Hydrochloric acid, sodium hydroxide, toluene, potassium dihydrogen phosphate, dipotassium hydrogen phosphate, sodium chloride, potassium chloride, and borax were supplied by Sinopharm Chemical Reagent Co., Ltd. (Shanghai, China). Poly(vinyl alcohol) (PVA) was purchased from Aladdin. *E. coli* was obtained from Shanghai Luwei Technology Co., Ltd. (Shanghai, China).

### 4.2. Instruments

Surface morphologies were examined using a scanning electron microscope (SEM, S-4800, Hitachi, Japan). Fourier transform infrared (FTIR) spectra were collected on a Bruker Vertex 70 FTIR spectrometer (Bruker Optics GmbH, Ettlingen, Germany). Thermogravimetric analysis (TGA) was performed using a TGA/SDTA851 analyzer (Mettler-Toledo, GmbH, Greifensee, Switzerland). Electrochemical measurements were carried out on a CHI760E electrochemical workstation (CH Instruments, Shanghai Chenhua Instrument Co., Ltd., Shanghai, China).

### 4.3. Preparation of the PVA/B Hydrogel

Poly(vinyl alcohol) (0.2 g) was dissolved in 1.2 mL of deionized water under stirring at elevated temperature in an oil bath for 3 h until complete dissolution. Subsequently, 0.3 mL of borax solution (0.01 mol L^−1^) was added dropwise under continuous stirring to form a borax-crosslinked PVA hydrogel (denoted as PVA-B). The resulting hydrogel was sterilized at 121 °C prior to use.

### 4.4. Construction of the PVA/B–A Bilayer Platform

The PVA/B–A bilayer platform was constructed by integrating an Agar-based cultivation layer with a borax-crosslinked PVA hydrogel detection layer. 0.1 g of nutrient agar medium was added to 9 mL of distilled water, heated until complete dissolution, and stirred to obtain a homogeneous solution. The obtained Agar solution was sterilized at 121 °C and then maintained in the molten state before use. Subsequently, the molten Agar solution was carefully dropped onto the upper surface of the preformed PVA/B hydrogel layer. After cooling and solidification, a PVA/B–Agar bilayer hydrogel platform, denoted as PVA/B–A, was obtained.

The resulting bilayer platform consisted of two functionally distinct modules. The upper Agar hydrogel layer served as the cultivation module to support the natural growth of *E. coli*, whereas the lower PVA/B hydrogel layer acted as the detection module for metabolite collection and electrochemical monitoring. During bacterial cultivation, purine-type metabolites secreted by *E. coli* diffused across the solid–solid interface from the Agar cultivation layer into the PVA/B detection layer, where they could be electrochemically detected.

For electrochemical monitoring, a conventional three-electrode system was inserted into the PVA/B hydrogel detection layer. A glassy carbon electrode (GCE, 3 mm in diameter) was used as the working electrode, a platinum wire as the counter electrode, and an Ag/AgCl electrode saturated with KCl as the reference electrode. Before use, the GCE was polished with 0.05 μm alumina slurry and rinsed thoroughly with distilled water. Cyclic voltammetry (CV) was performed to monitor the electrochemical behavior of *E. coli* metabolites in time-resolved over a potential range from 0.0 to +1.2 V at a scan rate of 90 mV·s^−1^.

### 4.5. Bacterial Culture and Plate Counting

Sterilized Agar medium was poured into Petri dishes and allowed to cool and solidify. The plates were then incubated at 37 °C for 24 h to confirm the absence of contamination. Subsequently, frozen *E. coli* stocks stored at −80 °C were taken out and thawed at 37 °C. An appropriate amount of bacterial suspension was streaked onto the Agar plates using an inoculation loop, followed by incubation at 37 °C for approximately 36 h. A single well-isolated colony was selected and transferred into sterile nutrient broth (NB) medium. After shaking incubation at 37 °C for about 6 h, the bacteria reached the exponential growth phase and were ready for subsequent antibiotic treatment.

Bacterial suspensions were serially diluted, and the diluted samples were spread onto sterile Agar plates. The plates were incubated at 37 °C for 24 h, after which the number of *E. coli* colonies was counted. When evaluating drug efficacy, the inhibition rate based on colony-forming units ($Inhibition_{CFU}$) was calculated as follows:

$$Inhibition_{CFU}\left(\%\right)=\left(1-N_{p}/N_{0}\right)\times 100\%$$

where $N_{0}$ represents the CFU in the absence of antibiotics, and $N_{p}$ represents the CFU in the presence of antibiotics.

### 4.6. Electrochemical Measurements and Quantitative Analysis

*E. coli* suspensions with different concentrations were uniformly inoculated onto the upper Agar cultivation module of the bilayer hydrogel detection system and incubated at 37 °C for 12 h prior to measurement. Electrochemical detection was performed under the following conditions: a scan rate of 90 mV·s^−1^, an accumulation time of 270 s, and an accumulation potential of 0 V. The change in peak current (ΔI) at the purine oxidation potential in the cyclic voltammograms (CVs) was recorded, and calibration plots were constructed by correlating ΔI with cell concentration to obtain the linear regression equation and correlation coefficient (R^2^). The limit of detection (LOD) was calculated based on a signal-to-noise ratio (S/N) of 3, and the accuracy and reproducibility of the method were evaluated using standard addition recovery experiments.

In addition, standard-addition experiments were performed to further validate the assignment of the purine-related oxidation signal in the actual bacterial matrix. After *E. coli* cultivation in the PVA/B–A platform, guanine (G), xanthine (X), and hypoxanthine (HX) standard solutions were added individually or as a mixed standard into the detection region of the hydrogel platform. After equilibration under the same conditions used for bacterial metabolite detection, CV measurements were performed using the optimized parameters described above. The oxidation peak current in the +0.80–0.90 V region was recorded before and after standard addition. The current increment after each addition was used to compare the relative responses of G, X, and HX in the *E. coli*-derived metabolic matrix and to support the assignment of the observed oxidation peak.

### 4.7. Time-Resolved Monitoring and Antibiotic Evaluation

For growth monitoring: *E. coli* suspensions (initial concentration: 75 × 10^6^ CFU·mL^−1^) were inoculated onto the upper agar cultivation module of the PVA/B-A platform at a volume of 75 μL. The platform was then incubated at 37 °C. Electrochemical measurements were performed at predetermined time intervals (1, 3, 5, 7, 9, 11, 13, 24, and 48 h) under optimized conditions (scan rate: 90 mV·s^−1^; accumulation potential: 0 V; accumulation time: 270 s). The signal–time curve was constructed based on the peak current changes (ΔI) at each time point to reflect the dynamic evolution of bacterial metabolism.

For antibiotic evaluation: The inhibitory effect of levofloxacin was evaluated by treating the bacteria in a liquid phase prior to platform inoculation. Specifically, *E. coli* suspensions (75 × 10^6^ CFU·mL^−1^) were mixed with various concentrations of levofloxacin (0–64 μg·mL^−1^) and incubated at 37 °C for 3 h. Subsequently, 75 μL of the drug-treated bacterial suspension was inoculated onto the upper agar layer of the PVA/B-A platform. The platform was incubated at 37 °C for an additional period to allow metabolites to diffuse into the detection module, followed by electrochemical interrogation. The electrochemical inhibition rate ($Inhibition_{EC}$) was calculated as follows:

$$Inhibition_{EC}\left(\%\right)=\left(1-\Delta I_{p}/\Delta I_{0}\right)\times 100\%$$

where $\Delta I_{0}$ and $\Delta I_{p}$ represent the electrochemical peak current responses (ΔI) in the absence and presence of levofloxacin, respectively. The resulting inhibition trends were compared with the $Inhibition_{CFU}$ data obtained from the plate counting method to validate the accuracy of the platform.

## Data Availability

Data will be made available on request.

## Supplementary Materials

The following supporting information can be downloaded at: https://www.mdpi.com/article/10.3390/gels12060538/s1, Figure S1: Dual-module detection system (**A**) reproducibility (*n* = 7) and (**B**) repeatability (*n* = 30). Guanine standard solution concentration: 1 µM. Scan rate: 90 mV/s; enrichment time: 270 s; enrichment potential: 0 V; Figure S2: Stability of the dual-module detection system. Guanine standard solution concentration: 1 µM. Scan rate: 90 mV/s; enrichment time: 270 s; enrichment potential: 0 V; Figure S3: Self-healing behavior of the PVA/B hydrogel; Figure S4: Electrochemical response of *Staphylococcus aureus* metabolites in the bilayer hydrogel platform; Figure S5: Quantitative evaluation of electrochemical signal recovery after repeated electrode puncture; Figure S6: Electrochemical impedance spectroscopy (EIS) analysis of the aqueous and PVA/B hydrogel systems; Table S1: Detection of *E. coli*-metabolized guanine (G) using the dual-module system. *E. coli* cell concentration: 7.5 × 10^8^ CFU; Table S2: Comparison Between PVA/B-A and the Latest Sensors [78,79,80,81,82].

## Abbreviations

The following abbreviations are used in this manuscript:

PVA	Poly(vinyl alcohol)
PVA/B	Borax-crosslinked poly(vinyl alcohol) hydrogel
A	Agar
PVA/B–A	PVA/B hydrogel and Agar bilayer platform
G	Guanine
X	Xanthine
HX	Hypoxanthine
CFU	Colony Forming Units
CV	Cyclic Voltammetry
GCE	Glassy Carbon Electrode
NB	Nutrient Broth
LAPS	Light Addressable Potentiometric Sensor
FET	Field-Effect Transistor
PCET	Proton-Coupled Electron Transfer
