# Supplementary material for: Self-Healing Bilayer Hydrogel Solid-State Electrochemical Platform: Time-Resolved In Situ Dynamic Monitoring of Escherichia coli Activity
Source: Gels. 2026 Jun 15;12(6):538. doi: 10.3390/gels12060538 (PMC13298827; doi:10.3390/gels12060538)
Supplement: Supplementary file 1 [file gels-12-00538-s001.zip › gels-4336209-supplementary.pdf]

Supplementary Materials

Self-Healing Bilayer Hydrogel Solid-State Electrochemical Platform: Time-Resolved In Situ Dynamic Monitoring of *Escherichia coli* Activity

Ye Li [1], Chaofan Zhang [1], Miao Zhang [1], Shi Zhou [2], Yanping Yu [3], Xiaoyan Yu [1], Ximing Cui [1,*] and Xiangge Qin [1,*]

[1] College of Materials Science and Engineering Technology, Jiamusi University, Jiamusi 154007, China; liye881006@163.com (Y.L.); yuxiaoyan@jmsu.edu.cn (X.Y.)

[2] College of Pharmacy, Jiamusi University, Jiamusi 154007, China; zhous146@nenu.edu.cn

[3] Jiamusi Center for Disease Control and Prevention, Jiamusi 154007, China; 15046463695@163.com

* Correspondence: cuiximing@jmsu.edu.cn (X.C.); qinxiangge@jmsu.edu.cn (X.Q.)

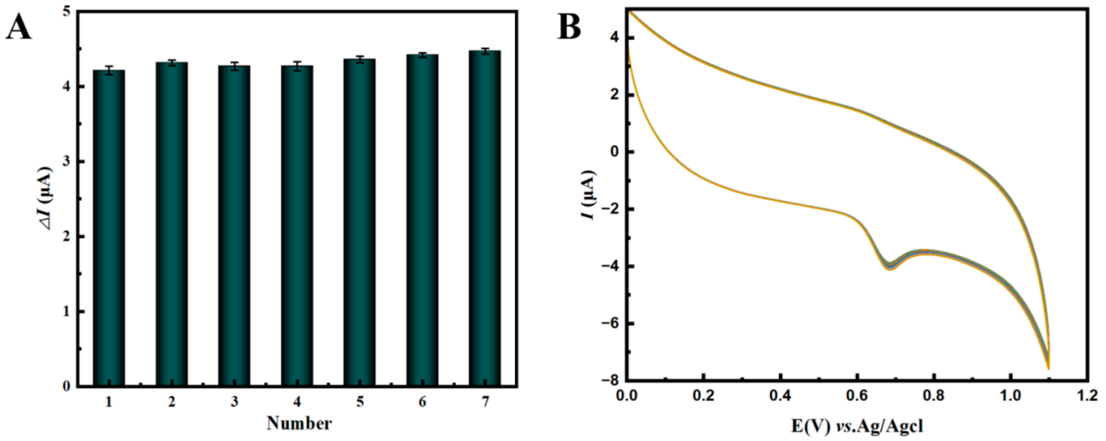

Figure S1: Dual-module detection system (**A**) reproducibility ($n = 7$) and (**B**) repeatability ($n = 30$). Guanine standard solution concentration: 1 μM. Scan rate: 90 mV/s; enrichment time: 270 s; enrichment potential: 0 V.

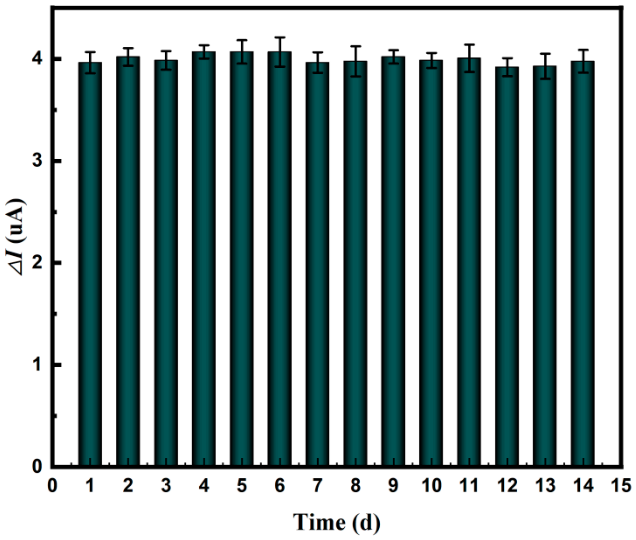

Figure S2: Stability of the dual-module detection system. Guanine standard solution concentration: 1 μM. Scan rate: 90 mV/s; enrichment time: 270 s; enrichment potential: 0 V.

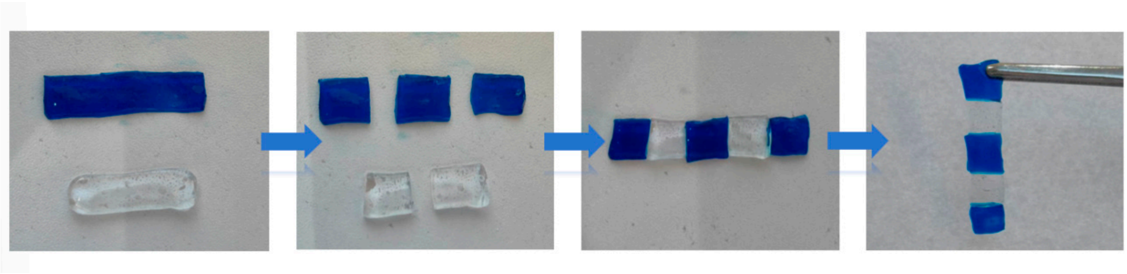

Figure S3: Self-healing behavior of the PVA/B hydrogel.

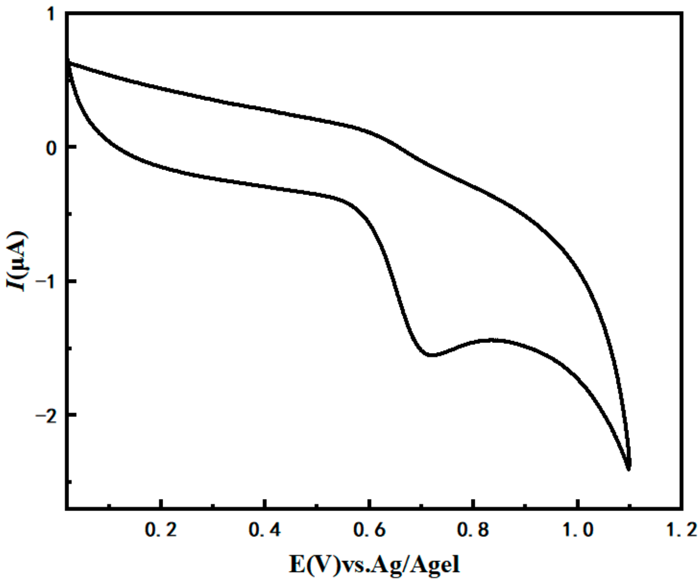

Figure S4: Electrochemical response of *Staphylococcus aureus* metabolites in the bilayer hydrogel platform.

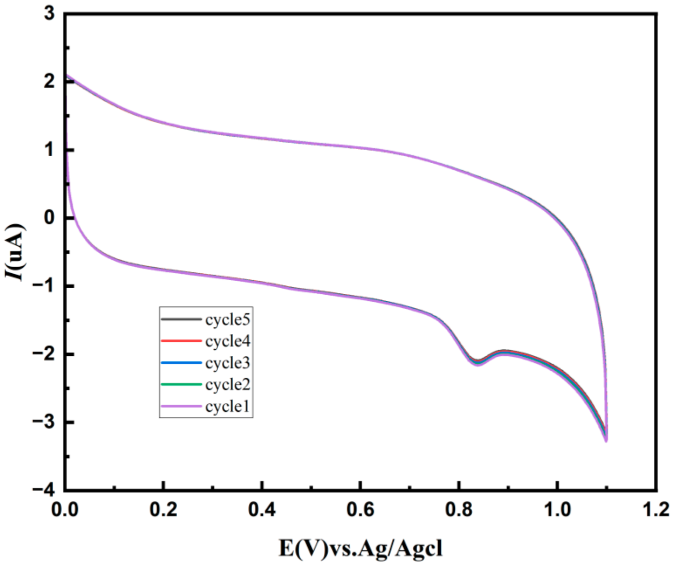

Figure S5: Quantitative evaluation of electrochemical signal recovery after repeated electrode puncture.

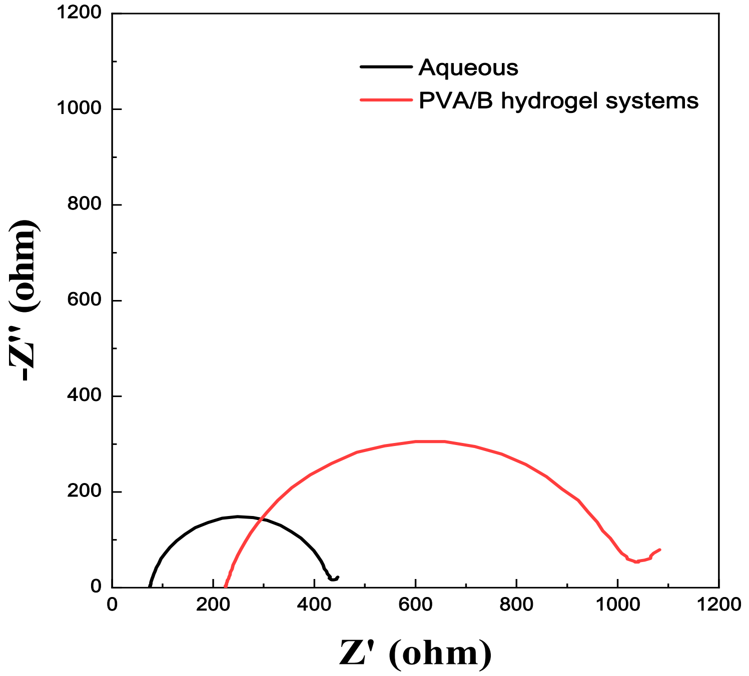

Figure S6: Electrochemical impedance spectroscopy (EIS) analysis of the aqueous and PVA/B hydrogel systems.

Table S1:

Detection of *E. coli*-metabolized guanine (G) using the dual-module system. *E. coli* cell concentration: 7.5 ×10^8 CFU.

Measured Value（μM）	Amount Added（μM）	Total Measured Value （μM）	Recovery Rate（%）
1.23 ± 0.04	0.5	1.71 ± 0.03	96.0
1.19 ± 0.05	1.0	2.13 ± 0.06	94.0
1.21 ± 0.03	1.5	2.62 ± 0.04	94.7
1.25 ± 0.06	2.0	3.28 ± 0.07	101.5
1.22 ± 0.02	3.0	4.25 ± 0.06	101.0

Table S2: Comparison Between PVA/B-A and the Latest Sensors

**Sensor/Platform**	**Detection mechanism**	**LOD (CFU mL^{-1})**	**Response time**	**in situ monitoring**	**Ref.**
Cationic covalent organic polymer thin-film sensor	Label-free impedimetric detection based on electrostatic capture of bacterial cells	2× 10^5	Not specified	No	[78]
Prussian blue-modified screen-printed carbon electrode	Receptor-free electrochemical monitoring of bacterial activity related to extracellular electron transfer	2 0	3 h	No	[79]
Bacterial-imprinted electrochemical sensor	Label-free recognition using bacterial-imprinted polypyrrole film	5	Not specified	No	[80]
Bacteria-imprinted polymer/ConA dual-recognition sensor	Sandwich-type recognition using bacteria-imprinted polymer and concanavalin A	10	Not specified	No	[81]
Vancomycin-assisted electrochemical aptasensor	Vancomycin-mediated bacterial capture combined with species-specific aptamer recognition	3.0 × 10^3	45 min	No	[82]
PVA/B–A bilayer hydrogel platform	Purine-related metabolic oxidation signal across a solid–solid hydrogel interface	3.0 × 10^5	5 min	Yes	This work
